# Determination of Anthelmintic and Antiprotozoal Drug Residues in Fish Using Liquid Chromatography-Tandem Mass Spectrometry

**DOI:** 10.3390/molecules26092575

**Published:** 2021-04-28

**Authors:** Eunjung Kim, Sihyun Park, Hyunjin Park, Jangduck Choi, Hae Jung Yoon, Jeong-Han Kim

**Affiliations:** 1 Pesticide and Veterinary Drug Residues Division, National Institute of Food and Drug Safety Evaluation, Ministry of Food and Drug Safety, Osong, Cheongju 28159, Korea; ejkim81@korea.kr (E.K.); sihyun11111@korea.kr (S.P.); hj77park@korea.kr (H.P.); cjd5388@korea.kr (J.C.); hjyoon@korea.kr (H.J.Y.); 2 Pesticide Chemistry and Toxicology Laboratory, Department of Agricultural Biotechnology and Research Institute of Agriculture and Life Sciences, Seoul National University, Seoul 08826, Korea

**Keywords:** anthelmintics, antiprotozoals, LC-MS/MS, residues, fishery product

## Abstract

The objective of this study is to develop a comprehensive and simple method for the simultaneous determination of anthelmintic and antiprotozoal drug residues in fish. For sample preparation, we used the “quick, easy, cheap, effective, rugged, and safe” (QuEChERS) method with a simple modification. The sample was extracted with water and 1% formic acid in acetonitrile/methanol (MeCN/MeOH) (95:5, *v*/*v*), followed by phase separation (salting out) with MgSO_4_ and NaCl (4:1, *w*/*w*). After centrifugation, an aliquot of the extract was purified by dispersive solid-phase extraction (d-SPE) prior to liquid chromatography-tandem mass spectrometry (LC-MS/MS) analysis. The method was validated at three concentration levels for all matrices, in accordance with the Codex guidelines (CAC/GL-71). Quantitative analysis was performed using the method of matrix-matched calibration. The recoveries were between 60.6% and 119.9%, with coefficients of variation (CV) <30% for all matrices. The limit of quantitation (LOQ) of the method ranged from 0.02 μg kg^−1^ to 4.8 μg kg^−1^ for all matrices. This comprehensive method can be used for the investigation of both anthelmintic and antiprotozoal drugs belonging to different chemical families in fishery products.

## 1. Introduction

Veterinary drugs and feed additives are widely used in animal farming to prevent and treat diseases, to protect the health of animals, and even as growth promoters. Many of these compounds, including antiparasitic agents such as anthelmintics and antiprotozoal drugs, are administered to prevent economic losses caused by disease in animal husbandry operations in many countries [1]. Anthelmintic drugs are one of the most widely used groups of veterinary drugs worldwide and are used to treat infections from roundworms (nematodes), tapeworms (cestodes), and flukes (trematodes). They are also essential for maintaining high weight and reproductive performance in livestock [2]. Drugs belonging to the chemical classes of macrocyclic lactones (avermectin, ivermectin, doramectin, and eprinomectin), benzimidazoles (albendazole, fenbendazole, and mebendazole), and flukicides (bithionol, clorsulon, closantel, and oxyclozanide), as well as individual compounds such as levamisole or morantel are considered to be anthelmintics. In aquaculture, praziquantels are used against intestinal tapeworms. Albendazole and fenbendazole are currently being investigated for use against flukes and larval tapeworms in salmon. Dichlovos and trichlorphon are organophosphates used to treat sealice infestation by the immersion bath method in the salmon industry [3].

Antiprotozoals are used to treat a variety of protozoal diseases. Triazine coccidiostats are benzene-aceto-nitrile compounds that have been used globally in veterinary drugs for years [4]. Moreover, triazine coccidiostats are widely used in livestock production because of their efficient broad-spectrum action and low toxicity [5,6,7]. Toltrazuril, ponazuril (toltrazuril sulfone), and diclazuril are triazine-based antiprotozoal drugs that have specific activity against coccidial infections. Nitroimidazoles are also antibacterial and antiprotozoal drugs used in veterinary and human medicine. Metronidazole (MET) is a nitroimidazole derivative primarily used to treat diseases caused by anaerobic bacteria and protozoa [8]. However, nitroimidazoles and their hydroxy metabolites that retain the original nitroimidazole ring are suspected to have carcinogenic, mutagenic, and toxic properties. [9,10,11]. For this reason, the use of MET, with other nitroimidazoles, has already been prohibited in the EU [12], South Korea [13], and other countries. Although this compound is prohibited as a food additive in many countries, it has the potential to be used in veterinary practice and aquaculture to achieve increased productivity and control parasitic infections [14].

Once administered, these antiparasitic agents are excreted either unchanged or as metabolites in urine or feces [15], and may retain their parasiticidal activity [16]. In some cases, the metabolite was found to be more toxic than the parent drug [17]. As a result, antiparasitic agents can have an impact on terrestrial and aquatic environments by direct excretion [18,19]. Once in the environment, these pollutants are transported to and distributed in the water. Some of these antiparasitic drugs are supplied through medicated feed. There is a strong possibility that the residues might make their way into fishery products by unintentional cross-contamination during storage and transportation or misuse during the manufacture of feed. It is clear that all these factors influence the presence of antiparasitic drug residues in aquatic animals. Therefore, it is necessary to develop efficient analytical methods that can monitor these drug residues in fish tissues, and thus ensure the safety of fishery products.

L.M. Rocca et al. [20] provided a comprehensive overview of veterinary drug residue analysis in various foodstuffs, where they discussed the latest analytical research on sample preparation and detection techniques. Based on this review and other published methods, liquid chromatography-tandem mass spectrometry (LC-MS/MS) is the most widely used technique for the simultaneous analysis of different veterinary drug groups in food matrices, owing to its high selectivity and sensitivity. The quick, easy, cheap, effective, rugged, and safe (QuEChERS) method has been successfully used for the sample preparation of a wide range of veterinary drug analytes and matrices. In addition, a modified QuEChERS-type extraction method was developed with an added concentration step [21,22]. Veterinary drug residues were extracted by acetonitrile (MeCN), using MgSO_4_ and NaCl to induce liquid–liquid partitioning, followed by a dispersive solid-phase extraction (d-SPE) clean-up step, by adding the supernatant into a centrifuge tube containing MgSO_4_ and octadecylsilane (C18). In addition, d-SPE can be performed using commercially available kits that reduce the extraction time, cost, solvent usage, and waste, and require less labor. In addition, minimal training is required. Several analytical methods have been described in the literature for the analysis of antiparasitic agents in feed [23,24], and in animal products and tissues, such as milk, liver, muscle, and egg [25,26,27,28,29,30]. However, most studies of anthelmintic and antiprotozoal residues have focused on livestock products and only a few studies report on mixtures of 20 or more types of drugs [27,31]. Moreover, multi-residue methods for the simultaneous determination of both anthelmintic and antiprotozoal drugs belonging to different chemical families in fish tissues are limited.

Therefore, the objective of this study is to develop, optimize, and validate a more comprehensive LC-MS/MS method for the determination of a wide range of antiparasitic agents and their transformation products in four matrices representative of fish tissues.

## 2. Results and Discussion

### 2.1. Optimization of Liquid Chromatography-Mass Spectrometry

The chromatographic conditions were optimized to achieve efficient separation, sensitivity, and selectivity, considering the different chemical properties of the target compounds. Chromatographic separation was achieved on a C18 column, which is widely used in drug analysis of livestock and fishery products [32]. Several mobile phases were tested to optimize the resolution and peak shape of the analytes. Three different versions of solvent A, the water phase, were tested as follows: (1) 0.1% formic acid and 2 mM ammonium formate in water, (2) 0.1% formic acid and 5 mM ammonium formate in water, and (3) 0.1% formic acid in water. Mobile phase 1 was found to be suitable for most analytes based on the shape of the peaks and the sensitivity. This result is consistent with those of the previous studies. When using formic acid in the aqueous phase, the most pronounced improvements included the shape of the peaks and separation. In addition, ammonium formate was included in the final mobile phase conditions, which significantly reduced peak tailing [27,33,34]. For solvent B (organic phase), 0.1% formic acid in MeCN was selected according to previously reported anthelmintic studies because it provides a better peak shape than that of methanol (MeOH) [35]. Under these conditions, the peaks were evenly distributed over a 20 min run time. Representative chromatograms of the target analytes obtained using a mixed standard solution are shown in Figure S1.

To optimize the mass spectrometry parameters, 71 standard solutions of each compound were directly injected into the MS. The electrospray source was used in positive and negative ion mode to provide the highest abundance. Three fragment ions per compound that gave highest abundance were selected; the first was used for quantification and identification, whereas others were used for confirmation. In this study, we used a positive/negative ion-switching method, which has the advantage of analyzing all analytes in a single run. In addition, other MS/MS parameters, such as declustering potential, cone voltage, and collision energy, were optimized using the automated quantitative optimization feature of the Labsolutions software. Most of the investigated compounds showed higher responses in positive ionization mode, and the remaining compounds appeared in negative ionization mode. The optimum conditions for mass spectrometry and the retention times for the investigated anthelmintic and antiprotozoal drugs are listed in Table S1.

### 2.2. Optimization of Sample Preparation Methods

The original QuEChERS method consists of two steps: an extraction/partitioning step with the addition of salts, and a clean-up step that uses d-SPE. In this study, a QuEChERS-based approach was adapted and optimized for sample extraction and clean-up procedures. The influence of several factors on the extraction and clean up of the analytes was evaluated by spiking blank flat fish samples at 50 μg kg^−1^. The extraction and clean-up efficiencies were investigated based on the number of drugs (% of total) and with consideration of the criteria recovery of 70–120% with coefficient of variation ≤20%.

#### 2.2.1. Extraction

The extraction efficiency was evaluated by analyzing flat fish muscle (2 g) using various extraction solvents: (1) 0.1% formic acid in MeCN/MeOH (95:5, *v*/*v*), (2) 0.1% formic acid in acetonitrile, and (3) 100% acetonitrile, followed by QuEChERS original powder (MgSO_4_: 4 g and NaCl: 1 g). The extraction efficiency results were as follows: (1) 88.3%, (2) 77.1%, and (3) 61.9%. The best recovery performance was obtained using 0.1% formic acid in MeCN/MeOH (95:5, *v*/*v*), followed by the original QuEChERS powder. We achieved results consistent with those from previous studies using a formic acid-acidified MeCN/MeOH extraction solution as the solvent to enhance the extraction of the drugs [34,36]. The inclusion of MeOH in the extraction solution indicated that it could increase the recovery of some compounds. However, the MeOH extracts were found to contain many matrix compounds. Thus, to obtain a cleaner extract, a MeCN/MeOH (95:5, *v*/*v*) solution was selected. Previous studies [37] reported that the overall drug recoveries using the QuEChERS methodology were lower than those obtained using liquid–liquid extraction (LLE). However, in the present study, a higher extraction efficiency was achieved for QuEChERS extraction than that for LLE using the same solvent.

#### 2.2.2. Clean-Up Procedure

The next step for sample preparation was to determine a suitable clean-up method by evaluating the effects of d-SPE extraction and liquid–liquid extraction(LLE). First, the recoveries of the following clean-up and extraction methods were compared: (1) d-SPE (containing PSA: 150 mg, C18: 150 mg, and MgSO_4_: 900 mg) clean-up, (2) MeCN saturated *n*-hexane extraction with the d-SPE clean-up, and (3) MeCN saturated *n*-hexane extraction. The results indicate that d-SPE extraction (80.2%) yielded better results than LLE with d-SPE extraction (44.9%) and LLE extraction (<10%). Subsequently, the influence of the extraction sorbents, primary secondary amine (PSA), and octadecylsilane (C18) was evaluated. We added MgSO_4_ under all conditions because of its ability to significantly reduce highly water-soluble polar matrix interferences, thereby improving the extraction efficiency of the samples. The original QuEChERS method uses PSA as a weak anion exchanger to effectively remove sugars and fatty acids from food samples [38]. C18 has been reported to allow the removal of non-polar compounds, including fats, oil, and pigments [39]. PSA achieved the highest analyte recoveries; however, it yielded lower recovery and poor reproducibility in the case of fluazuron and buparvaquone. C18 afforded lower recoveries of oxantel, amprolium, and thiophanate. A mixture of PSA and C18 was also evaluated, but afforded a lower recovery than PSA alone. However, this combination was selected as the most suitable sorbent for the method because of its ability to provide effective clean up while achieving satisfactory recoveries for most analytes.

Finally, the re-constitution solvent of the extract was investigated to improve the signal intensity of the compounds. The extract was evaporated under a gentle nitrogen stream and reconstituted with various solvent mixtures: (1) MeOH/aqueous solution (50:50, *v*/*v*), (2) 0.1% formic acid in MeOH/aqueous solution (50:50, *v*/*v*), and (3) 0.1% formic acid in 1 mM ammonium formate/MeCN solution. The MeOH/aqueous solution (79.8%) produced better results than the 0.1% formic acid in MeOH/aqueous solution (47.1%) and 0.1% formic acid in 1 mM ammonium formate/MeCN solution (67.3%). Dimethylsulfoxide (DMSO) was added to the sample extract before concentration because it acts as a keeper during evaporation and reduces the potential for protein binding [40].

In summary, the optimized sample preparation method comprised two preparation steps: (1) sample extraction with 0.1% formic acid in MeCN/MeOH (95:5, *v*/*v*) and the original QuEChERS salts, and (2) clean up with d-SPE including PSA and C18 sorbent. The dissolved solution was MeOH/aqueous solution (50:50, *v*/*v*) with DMSO.

### 2.3. Method Validation

#### 2.3.1. Validation of Analytical Method

The method was validated according to the Codex guidelines (CAC/GL 71-2009) [41]. The evaluation parameters were selectivity, linearity of calibration, accuracy, precision, limit of quantitation (LOQ), and matrix effects. In LC-MS/MS, the criteria for the relative retention times and ion ratios were examined for all samples and standards used for the validation. We investigated whether these values were consistent with the CODEX requirements for all analyses. The selectivity was evaluated by determining whether there was interference with the spiked target compounds in different matrices. The chromatograms of the spiked sample solution and standard mixture solution of the target analytes were compared. The linear regression coefficients (R^2^) for a six-point method matrix-matched calibration were >0.98 for all compounds within the target ranges. The accuracy and precision of the established method were evaluated for each of the target compounds using the average and the coefficient of variation (CV, %) of recovery (*n* = 5). The results of the recovery and precision experiments are shown in Table 1 and Table S3.

**Figure 1:**
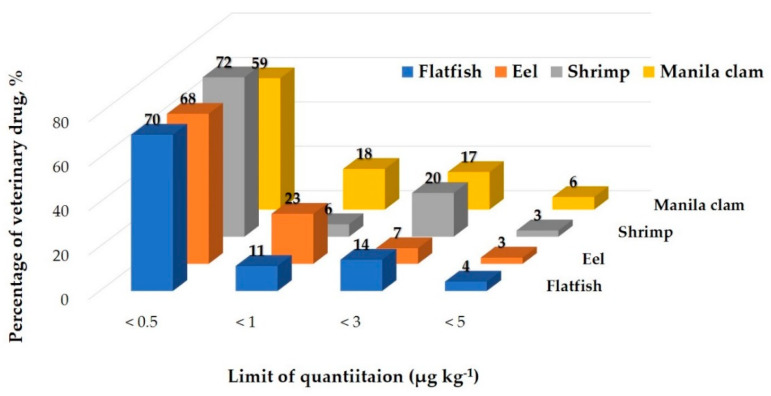
Distribution of the limit of quantitation.

All analytes met the Codex validation requirements with recovery values in the range of 60–119%, while the CV ranged from 0.3 to 30%. Therefore, this modified QuEChERS method was shown to be rapid and reliable for the quantification of the target compounds in fishery products. Some substances demonstrated CV values of 20 or higher at low concentrations (5 μg kg^−1^). The matrix that contained the most substances with a CV value of 20 or higher was eel. This was believed to be because of the high fat content of eel. Keto triclabendazole produced high CV values in all four matrices. This is consistent with the results of previous studies [42]. However, the three verified concentrations did not all produce high CV values. The only substances with CV values above 20 at all three verified concentrations were triclabendazole in Manila clam and narasin in eel. Nevertheless, the CV value achieved in the experiment satisfies the Codex Guideline criteria (≤30). The LOQ for all 71 compounds ranged from 0.02 μg kg^−1^(albendazole, buquinolate, and monepantel) to 4.8 μg kg^−1^(clorsulon) in the four matrices (Figure 1 and Table S2). In the LOQ distribution (Figure 1), more than half of the target veterinary drugs (70% of the total in flatfish, 68% of the total in eel, 72% of the total in shrimp and 59% of the total in Manila clam) showed LOQ ≤0.5 μg kg^−1^. The LOQs of clorsulon, dicyclanil, and oxfendazole were slightly higher in all matrices. Otherwise, the LOQ values of all other analytes in this study are similar or lower than those from previous studies. M.E. Dasenaki et al. [32] reported that LOQs of 115 veterinary drugs were in all cases below 5 μg kg-1 in fish tissue and R.P. Lopes et al. [36] reported that LOQs of 32 veterinary drugs were lower than 25 μg kg^−1^ in aquaculture fish.

#### 2.3.2. Matrix Effect

Quantitative analysis with electrospray ionization (ESI) can be substantially affected by ion suppression or enhancement caused by matrix or other interferences in the sample [43]. This phenomenon is commonly referred as “matrix effects (MEs)”, and can lead to erroneous quantitation. Therefore, the MEs were tested and evaluated during validation. To evaluate the MEs, the results were classified into three groups: soft effect (matrix effects between −20% and 0% or 0% and 20%), medium effect (−50% and −20% or 20% and 50%), and strong effect (<−50% or >50%) [44].

**Figure 2:**
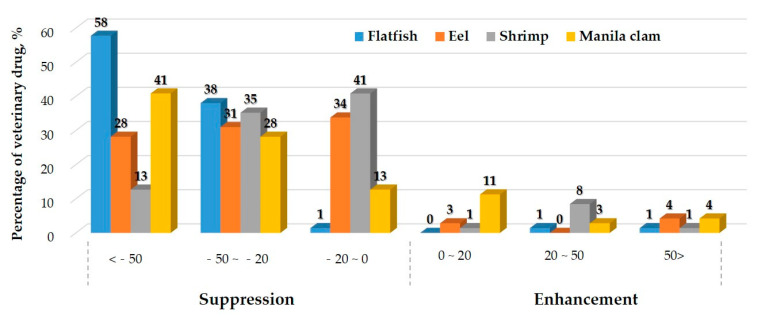
Distribution of matrix effects.

The MEs calculated for all the compounds in each matrix are listed in Table S2 and Figure 2. Because of the unpredictability of MEs, variations in the responses can be observed even among different samples using the same method. According to the matrices, the matrix effects were mostly soft and medium in eel and shrimp, but medium and strong effects were observed in flat fish and Manila clams. Strong effects were observed for 41 compounds in flat fish (59.2% of total), 24 compounds in eel (32.4% of total), 10 compounds in shrimp (14.1% of total), and 31 compounds in Manila clam (45.1% of total). Amprolium, buparvaquone, closantel, dicyclanil, fluazuron, and 5-Hydroxy thiabendazole presented strong suppression in all matrices. Most of the compounds in the fish tissue were subjected to signal suppression, whereas few compounds were subjected to signal enhancement. Monepantel, and monepantel-sulfone presented signal enhancement in all matrices. The greatest suppression in all matrices was 99% (ME −99%) for thiophanate in Manila clam, while the highest enhancement was observed for Monepantel-sulfone (ME +111%) in Manila clam. Careful sample clean-up procedures and more efficient chromatographic separations can reduce the interference of co-eluting matrix compounds. However, this approach can be quite time-consuming and requires a significant effort and can lead to analyte loss. The use of isotope-labeled internal standards (ISs) has also been employed; nevertheless, it is worth noting that labeled standards are expensive and not available for all compounds [45]. When multi-residue analysis is performed, the use of ISs to reduce the MEs can be unsatisfactory because of the large number of compounds with a wide range of physicochemical properties [46]. Therefore, the matrix-matched calibration method probably represents the most effective way to compensate for MEs [47], and we used it to improve the method performance in this study.

### 2.4. Application to Real Samples

The established method was applied to 35 real samples to determine and quantify veterinary drugs in fishery products in South Korea. Several species of fish (flat fish, eel, and shrimp) were collected from the South Korean domestic market and analyzed to test the method. According to the results, no compounds were present in the samples. The results were reasonable, as only three drugs (bithionol, praziquantel, and trichlorfon) are approved for use in aquaculture in South Korea. In addition, this result implies that there was no use of drugs that are banned in fisheries, which confirmed that veterinary drugs are being used safely in South Korea. However, our monitoring results have limitations in determining anthelmintic and antiprotozoal drug residues in fishery products. Therefore, further studies are required to determine the residues in a large number of fish samples and to apply them to various matrices such as bovine, egg, and milk.

## 3. Materials and Methods

### 3.1. Chemicals, Standards, and Stock Solutions

All high purity grade (>90%) chemical standards were purchased from Sigma-Aldrich (St. Louis, MO, USA); these include 5-hydroxy thiabendazole, albendazole sulfoxide, bithionol, carbendazim, fluazuron, keto triclabendazole, maduramycin, oxamniquine, ternidazole, thiophanate, and toltrazuril sulfone. 22,23-Dihydroavermectin b1a (ivermectin), avermectin b1a (abamectin), arprinocid, benznidazole, buparvaquone, diethylcarbamazine, halofuginone, and zoalene were purchased from Toronto Research Chemicals (Toronto, ON, Canada). Emamectin b1a (emamectin) and monepantel were purchased from ChemService (West Chester, PA, USA). Narasin and monepantel-sulfone were purchased from USP (Rockville, MD, USA) and HPC Standards GmbH (Cunnersdorf, Germany), respectively. Ornidazole was purchased from Stordsynthesis (Hebei Province, China), and the remaining standards were purchased from Dr. Ehrenstorfer (Augsburg, Germany). HPLC-grade MeOH and MeCN were purchased from Merck Inc. (Darmstadt, Germany). DMSO, formic acid, MgSO_4_, and NaCl were purchased from Sigma-Aldrich (St. Louis, MO, USA) and PSA was purchased from Agilent Technologies (Santa Clara, CA, USA). Other reagents and solvents, such as ammonium formate, were purchased from Alfa Aesar (Ward Hill, MA, USA), and C18 (55–105 μm, 125 Å) was purchased from Waters (Milford, MA, USA). We purchased 0.2 μm polytetrafluoroethylene (PTFE) filter from Teknokroma (Barcelona, Spain). Standard stock solutions (1000 μg mL^-1^) were prepared for each compound in MeCN, MeOH, MeOH/DMSO (1:1, v/v), and DMSO. All standard stock solutions were stored in the dark at −20 °C.

### 3.2. Sample Collection and Preparation

We collected three species (*n* = 35), viz., flat fish (*n* = 12), eel (*n* = 11), and shrimp (*n* = 12), from fish markets and online fish markets in South Korea. They were homogenized and stored in a freezer at −20 °C until use in the experiment. A portion of the homogenized samples (2 g) was weighed and placed in a 50 mL centrifuge tube. For extraction, 10 mL of 1% formic acid in MeCN/MeOH (95:5, *v*/*v*) and 10 mL of water were added and mixed with the sample for 5 min. Next, the original QuEChERS salt (4 g of MgSO_4_ and 1 g of NaCl) was added to the sample, shaken for 5 min, and then centrifuged at 4700× *g* for 10 min at 4 °C. The supernatant was transferred to a 15 mL centrifuge tube containing 150 mg of C18, 150 mg of PSA, and 900 mg of MgSO_4_. The mixture was shaken for 5 min and then centrifuged at 4700× *g* for 5 min at 4 °C. Then, a 5 mL aliquot of the supernatant was transferred to another tube, and 20 μL of DMSO was added. The sample was completely evaporated under a stream of nitrogen at 40 °C. The residue was then dissolved in 1 mL of MeOH/water (1:1, *v*/*v*) and filtered through a 0.2 μm PTFE filter before analysis.

### 3.3. LC-MS/MS Conditions

Chromatography was performed using a Shimadzu LCMS 8060 triple quadrupole mass spectrometer (Shimadzu, Kyoto, Japan) with an X-SELECT HSS C18 column (2.1 mm × 150 mm, 3.5 μm, Waters, Dublin, Ireland) at 40 °C. The mobile phase for the positive/negative mode analysis was 0.1% formic acid in 2 mM ammonium formate in water (A) and 0.1% formic acid in MeCN (B). The gradient mode proceeded accordingly: starting at 0 min, B increased to 15%, B increased further to 95% from 2 to 12.5 min, then remained at 95% from 12.5 to 17 min, decreased to 15% from 17 to 17.1 min, and finally remained at 15% from 17.1 to 20 min. The flow rate was 0.3 mL min^−1^ and the injection volume was 5 µL. The total chromatographic run time was 20 min. Mass spectrometry was performed using an electrospray ionization (ESI) source in both positive and negative switching modes. The capillary and autosampler temperatures were maintained at 350 °C and 15 °C, respectively. Capillary voltages were 4.0 kV (positive) and −2.8 kV (negative), the cone voltage was 30 kV for all compounds, and argon gas was used. The scheduled multiple reaction monitoring (MRM) was applied to all target compounds, and Table 1 lists the detailed parameters for the MRM acquisition.

### 3.4. Method Validation and Matrix Effect

The developed method was validated according to the procedures described in the Codex guidelines (CAC/GL-71). We evaluated the performance of parameters, such as accuracy, precision, selectivity, linearity, repeatability, matrix effect, and limit of quantitation (LOQ). Accuracy and precision were expressed as the recovery and coefficient of variation (CV). The recoveries of most compounds were tested by spiking blank samples at three different concentrations and estimated from five replicates for each concentration: 0.5, 1, and 2 times the target concentration (TC), set at 10 μg kg^−1^. Metronidazole, an unauthorized compound, was validated at 1, 2, and 10 times 1 μg kg^−1^. All concentration levels used in the validation study are listed in Table 1 and S2. The selectivity of the method was compared through extracted blank samples, and the blank samples of spiked target compounds. The matrix-matched calibration curves were prepared by fortifying matrix blanks before extraction with working standard mixes, prepared at the following concentrations at six different concentrations (metronidazole: 0.5, 1, 2, 10, 20, and 40 μg kg^-1^; and other compounds: 2.5, 5, 10, 20, 40, and 80 μg kg^−1^). These extracted matrix-matched calibration curves were used to obtain the validation data. Moreover, the following compounds were analyzed according to the marker residue given in parentheses: Abamectin (avermectin B1a), emamectin (emamectin B1a), ivermectin (22,23-dihydroavermectin B1a), and nicarbazin (N,N’-bis(4-nitrophenylurea)). The LOD and LOQ were defined with signal-to-noise (S/N) ratios of ≥3 and ≥10, respectively. The LOQs were calculated by analyzing blank samples spiked at the lowest concentration of the analyte for which the signal-to-noise ratio was 10.

The degree of matrix effect was estimated by comparing the signal of the analyte in a standard solution to that of a post-extraction spiked sample at the same concentration (matrix-matched standard) and can be expressed using Equation (1).

(1) $$\mathrm{Matrix}\;\mathrm{Effect}\left(\%\right)=\left(\frac{Slope{\;}_{of\;calibration\;in\;matrix\;matched\;standard\;}}{Slope_{\;of\;calibration\;in\;solvent\;standard}}-1\right)\times 100$$

## 4. Conclusions

The described method is suitable for the simultaneous analysis of anthelmintic and antiprotozoal drugs in fish products by LC-MS/MS with good linearity, selectivity, and repeatability as well as with LOQs that comply with the established maximum residue levels (MRLs). The multi-class method covers substances from 71 different veterinary drugs and comprises simple sample preparation and analysis in one measurement by LC-MS/MS. A solution of water and MeCN was selected to dissolve a wide range of analytes with different physicochemical properties. The extraction process was evaluated by modifying the QuEChERS approach to provide comprehensive and more effective extraction. The method was validated in a single laboratory according to the Codex guidelines and was designed to meet the acceptability criteria. Furthermore, the developed method was applied to investigate the levels of anthelmintic and antiprotozoal drugs in fish, eel, and shrimp samples in South Korea. Until now, monitoring of antiparasitic agents in aquatic products has been conducted only for compounds that have established MRLs. Therefore, this method can be an efficient means of regulatory monitoring for residue quantification of a variety of veterinary drugs in fish.

## Figures and Tables

**Table 1:** Summary of method validation parameters; recovery and coefficient value (CV) for the 71 target veterinary drugs (*n* = 5).

Recovery	Number of Veterinary Drugs (Percentage, %)			
	Flatfish	Eel	Shrimp	Manila Clam
**L1**	(CV 2.3%~28.4%)	(CV 2.8%~30.2%)	(CV 0.8%~26.6%)	(CV 1.7%~29.7%)
60% to 80%	3(4.2%)	7(9.9%)	11(15.5%)	5(7.0%)
80% to 100%	38(53.5%)	35(49.3%)	35(49.3%)	30(42.3%)
100% to 120%	30(42.3%)	29(40.8%)	25(35.2%)	36(50.7%)
**L2**	(CV 1.7%~23.1%)	(CV 1.5%~24.6%)	(CV 1.1%~14.2%)	(CV 2.2%~27.6%)
60% to 80%	1(1.4%)	1(1.4%)	0(0.0%)	2(2.8%)
80% to 100%	24(33.8%)	29(40.8%)	38(53.5%)	31(43.7%)
100% to 120%	46(64.8%)	41(57.7%)	33(46.5%)	38(53.5%)
**L3**	(CV 0.9%~18.8%)	(CV 1.5%~20.4%)	(CV 1.0%~17.5%)	(CV 1.8%~22.2%)
60% to 80%	1(1.4%)	1(1.4%)	0(0.0%)	1(1.4%)
80% to 100%	33(46.5%)	37(52.1%)	44(62.0%)	11(15.5%)
100% to 120%	37(52.1%)	33(46.5%)	27(38.0%)	59(83.1%)

## Data Availability

Data are contained within the article or the Supplementary Materials.

## Supplementary Materials

The following are available online, Table S1. LC-MS/MS parameters of 71 compounds. Table S2. Linearity, matrix effects, and LOQs of 71 compounds. Table S3. Validation results for the analytical method of 71 compounds in 4 kinds of food matrices. Figure S1. Representative chromatograms in flatfish at the spiking levels of 10 μg kg^-1^.

## Sample Availability

Samples of the compounds are not available from the authors.
