# Supplementary material for: Determination of Anthelmintic and Antiprotozoal Drug Residues in Fish Using Liquid Chromatography-Tandem Mass Spectrometry
Source: Molecules. 2021 Apr 28;26(9):2575. doi: 10.3390/molecules26092575 (PMC8125621; doi:10.3390/molecules26092575)
Supplement: Supplementary file 1 [file molecules-26-02575-s001.zip › molecules-1191395-supplementary.pdf]

molecules

MDPI

Supplementary Material

Determination of Anthelmintic and Antiprotozoal Drug Residues in Fish Using Liquid Chromatography-Tandem Mass Spectrometry

Eunjung Kim [1,2,†], Sihyun Park [1,†], Hyunjin Park [1], Jangduck Choi [1], Hae-Jung Yoon [1] and Jeong-Han Kim [2,*]

[1] Pesticide and Veterinary Drug Residues Division, National Institute of Food and Drug Safety Evaluation, Ministry of Food and Drug Safety, Osong, Cheongju, Chungcheongbuk-do, 28159, Korea; ejkim81@korea.kr (E.K.); sihyun11111@korea.kr (S.P.); hj77park@korea.kr (H.P.); cjd5388@korea.kr (J.C.); hjyoon@korea.kr (H.-J.Y.)

[2] Pesticide Chemistry and Toxicology Laboratory, Department of Agricultural Biotechnology and Research Institute of Agriculture and Life Sciences, Seoul National University, Seoul, 08826, Korea

* Correspondence: kjh2404@snu.ac.kr; Tel.: +82-2-880-4644

† These authors contributed equally to this work.

Table S1. LC-MS/MS parameters of 71 compounds.

Class	Compounds	ESI (+/−)	Molecular weight (*m*/*z*)	Precursor ion (*m*/*z*)	[a]Product ion (*m*/*z*)	Collision Energy (eV)	Retention time (min)
Anthelmintic	Abamectin	+	872.5	895.0	**449.3**	20	13.76
					327.3	20	
					751.4	20	
	Albendazole	+	265.1	266.0	**234.0**	20	8.00
					191.1	20	
					159.2	20	
	Albendazole sulfoxide	+	281.1	282.0	**208.1**	16	5.12
					240.1	19	
					159.2	19	
	Albendazole sulfone	+	297.1	298.0	**159.2**	20	6.19
					224.1	20	
					266.1	20	
	2-Amino albendazole sulfone	+	239.1	240.0	**133.2**	17	2.84
					198.2	16	
					105.2	30	
	Benznidazole	+	260.1	261.0	**91.2**	20	6.51
					107.3	18	
					65.3	17	
	Bithionol	−	353.9	352.0	**161.1**	−25	12.43
					192.0	−25	
					125.1	−42	
	Carbendazim	+	191.1	192.0	**132.2**	20	2.78
					105.2	30	
					160.1	30	
	Carnidazole	+	244.1	245.0	**118.2**	30	6.51
					75.2	14	
					47.2	26	
	Clorsulon	−	378.9	377.0	**342.1**	−14	6.87
					242.0	−24	

Closantel	−	661.9	660.0	142.2	−29	13.87
				**126.9**	−32	
				315.1	−34	
				344.9	−24	
Cymiazole	+	218.1	219.0	**171.2**	15	12.68
				144.1	20	
				77.3	23	
Diethylcarbamazine	+	199.2	200.0	**100.0**	20	1.79
				72.0	20	
				44.2	20	
Doramectin	+	898.5	921.0	**777.4**	36	14.38
				449.3	34	
				353.3	34	
Emamectin	+	885.5	886.0	**158.1**	30	10.98
				82.2	34	
				159.2	32	
Febantel	+	446.1	447.0	**325.2**	13	10.50
				93.2	25	
				384.1	16	
Fenbendazole	+	299.1	300.0	**416.2**	17	8.90
				281.2	30	
				268.0	20	
Fluazuron	+	505.0	506.0	**95.2**	17	12.50
				75.3	30	
				141.2	34	
Flubendazole	+	313.1	314.0	**83.3**	22	7.90
				203.0	22	
				282.1	34	
2-Amino flubendazole	+	255.1	256.0	**123.2**	34	6.00
				95.2	34	
				123.2	17	
Ivermectin	+	874.5	897.0	**753.4**	40	15.62
				329.3	34	
				609.3	24	
Levamisole	+	204.1	205.0	**91.0**	20	2.91
				123.1	20	
				178.0	20	
Mebendazole	+	295.1	296.0	**264.0**	20	7.55
				105.2	30	
				77.2	20	
Mebendazole amine	+	237.1	238.0	**105.2**	16	5.71
				133.3	30	
				77.2	16	
5-Hydroxy mebendazole	+	297.1	298.0	**266.0**	20	5.88
				79.2	20	
				160.2	20	
Monepantel	−	473.1	472.0	**186.0**	−20	11.57
				166.2	−23	
				146.2	−14	
Monepantel sulfone	−	505.1	504.0	**186.0**	−30	11.05
				166.1	−24	
				146.1	−24	

Morantel	+	220.1	221.0	**123.0**<br>164.0<br>111.0	20<br>20<br>21	5.58
Niclosamide	−	326.0	325.0	**171.1**<br>289.0<br>135.1	−20<br>−18<br>−21	11.22
Nitroxinil	−	289.9	288.0	**127.0**<br>162.1<br>116.1	−14<br>−28<br>−20	7.79
Ornidazole	+	219.0	220.0	**82.3**<br>128.2<br>42.2	20<br>10<br>10	5.29
Oxamniquine	+	279.2	280.0	**221.1**<br>174.2<br>60.3	20<br>20<br>20	3.16
Oxantel	+	216.1	217.0	**91.1**<br>118.3<br>131.3	20<br>20<br>29	3.08
Oxfendazole	+	315.1	316.0	**159.2**<br>284.2<br>191.2	19<br>21<br>21	6.23
Oxfendazole sulfone	+	331.1	332.0	**300.1**<br>159.2<br>131.3	19<br>12<br>12	7.23
Oxibendazole	+	249.1	250.0	**176.0**<br>218.0<br>148.2	20<br>20<br>18	6.57
Oxyclozanide	−	398.9	397.0	**362.0**<br>202.0<br>176.2	−19<br>−24<br>−27	11.08
Praziquantel	+	312.2	313.0	**159.0**<br>131.3<br>174.2	20<br>21<br>19	8.97
Pyrantel	+	206.1	207.0	**158.2**<br>351.1<br>109.0	34<br>34<br>20	4.25
Ternidazole	+	185.1	186.0	**82.2**<br>128.2<br>42.2	30<br>12<br>12	2.87
Tetramisole	+	204.1	205.0	**178.0**<br>91.0<br>123.1	20<br>20<br>20	2.91
Thiabendazole	+	201.0	202.0	**121.2**<br>175.0	19<br>20	3.20
5-Hydroxy thiabendazole	+	217.0	218.0	**131.0**<br>65.2<br>191.1	20<br>26<br>16	1.92
Thiophanate	+	370.1	371.0	**147.2**<br>81.3<br>151.0	16<br>15<br>20	9.11
Trichlorfon	+	255.9	256.0	**109.2**	17	5.28

					221.1	11	
					113.0	31	
					**273.9**	35	
	Triclabendazole	+	358.0	359.0	343.9	30	10.99
					171.1	53	
					**182.1**	−25	
	Keto triclabendazole	−	328.0	326.9	146.1	−34	9.63
					118.0	−40	
					**142.9**	20	
Antiprotozoal	Arprinocid	+	277.1	278.0	107.2	16	6.01
					108.2	19	
					**150.0**	20	
	Amprolium	+	278.1	243.0	122.1	20	1.07
					94.1	22	
					**186.1**	−30	
	Buparvaquone	−	326.2	325.0	297.3	−21	14.47
					187.2	−12	
					**204.0**	20	
	Buquinolate	+	361.2	362.0	316.1	21	10.05
					148.2	25	
					**217.0**	20	
	Cambendazole	+	302.1	303.0	261.0	20	6.15
					190.1	24	
					**101.2**	26	
	Clopidol	+	191.0	192.0	110.2	12	2.34
					128.2	26	
					**372.1**	20	
	Decoquinate	+	417.3	418.0	204.1	27	12.68
					232.2	15	
					**123.1**	20	
	Diaveridine	+	260.1	261.0	81.3	20	2.84
					245.0	19	
					**334.1**	−26	
	Diclazuril	−	406.0	405.0	335.1	−28	10.49
					**41.2**	14	
	Dicyclanil	+	190.1	191.0	150.3	14	0.56
					109.2	13	
					**136.1**	20	
	Ethodpabate	+	237.1	238.0	80.2	20	6.81
					206.0	27	
					**136.0**	20	
	Halofuginone	+	413.0	414.0	150.0	20	6.19
					100.2	29	
					**188.2**	20	
	Imidocab	+	348.2	349.0	162.2	13	1.54
					97.7	24	
					**393.4**	22	
	Maduramycin	+	916.5	935.0	647.5	26	16.85
					629.4	22	
	Methylbenzoquate, Nequinate	+	365.2	366.0	**334.1**	20	9.88
					91.0	22	

				201.1	26	
				**82.2**	12	
Metronidazole	+	171.1	172.0	42.1	12	2.28
				128.2	11	
				**675.4**	24	
Monensin	+	692.4	693.0	461.4	24	16.60
				479.3	24	
				**531.4**	36	
Narasin	+	764.5	787.0	431.3	30	17.32
				769.5	30	
				**136.9**	−20	
Nicarbazin	−	302.1	301.0	107.1	−30	9.74
				46.0	−20	
				**432.3**	28	
Salinomycin	+	772.5	773.0	431.3	28	16.13
				532.3	28	
				**629.4**	34	
Semduramicin	+	872.5	890.0	647.4	20	14.49
				727.5	34	
				**128.2**	11	
Tinidazole	+	247.1	248.0	82.2	11	4.30
				121.2	19	
				**41.8**	−22	
Toltrazuril sulfone	−	457.1	456.0	399.1	−22	9.96
				**42.1**	−15	
Zoalene	−	225.0	224.0	77.1	−27	6.12
				181.1	−23	

[a] The bold underlined text expressed as quantification ion.

Table S2. Linearity, matrix effects and LOQs of 71 compounds.

No.	Compound	Flatfish			Eel			Shrimp		
		Linearity (R^2)	ME[a] (%)	LOQ[b] (μg kg^{-1})	Linearity (R^2)	ME[a] (%)	LOQ[b] (μg kg^{-1})	Linearity (R^2)	ME[a] (%)	LOQ[b] (μg kg^{-1})
1	Abamectin	0.9962	−17.8	0.3	0.9967	1.1	0.3	0.9872	47.0	0.2
2	Albendazole	0.9826	−55.8	0.02	0.9826	−45.0	0.1	0.9921	−10.7	0.1
3	Albendazole sulfoxide	0.9969	−10.4	0.1	0.9952	3.9	0.1	0.9992	−11.0	0.2
4	Albendazole sulfone	0.9992	−38.3	0.1	0.9957	−45.7	0.1	0.9954	−20.6	0.1
5	2-Amino albendazole sulfone	0.9992	−22.9	0.3	0.9983	−30.9	0.4	0.9978	−19.8	0.1
6	Arprinocid	0.9997	−54.0	0.2	0.9996	−19.2	0.2	0.9993	−19.8	0.2
7	Amprolium	0.9855	−92.7	1.8	0.9946	−83.6	3.6	0.9945	−94.9	3.9
8	Benznidazole	0.9994	−42.3	1.7	0.9995	−0.1	0.8	0.9972	−11.4	0.3
9	Bithionol	0.9916	−60.3	0.4	0.9871	−71.0	0.2	0.9963	−47.6	0.2
10	Buparvaquone	0.9984	−76.5	0.1	0.9810	−83.6	0.5	0.9897	−62.6	0.2
11	Buquinolate	0.9916	−47.6	0.02	0.9927	−65.8	0.3	0.9983	−19.5	0.1
12	Cambendazole	0.9996	−38.6	0.1	0.9972	−19.8	0.3	0.9965	−6.7	0.1
13	Carbendazim	0.9998	−61.3	0.6	0.9988	−27.1	0.7	0.9993	−37.0	0.7
14	Carnidazole	0.9953	−43.9	1.2	0.9992	−23.4	1.2	0.9983	−2.2	1.2

15	Clopidol	0.9962	−59.3	0.2	0.9972	−18.5	0.2	0.9997	−33.5	0.1
16	Clorsulon	0.9983	−24.1	3.3	0.9833	−4.9	2.7	0.9983	−22.9	2.6
17	Closantel	0.9880	−51.1	0.1	0.9840	−52.6	0.03	0.9853	−93.3	0.1
18	Cymiazole	0.9938	−52.0	0.5	0.9989	−38.3	0.1	0.9983	−23.3	2.2
19	Decoquinate	0.9931	−73.6	0.1	0.9987	−95.5	0.1	0.9949	−37.9	0.1
20	Diaveridine	0.9972	−28.8	0.3	0.9991	−15.8	0.4	0.9954	−22.5	0.5
21	Diclazuril	0.9893	−53.5	0.2	0.9994	−39.1	0.1	0.9902	−23.0	0.1
22	Dicyclanil	0.9953	−86.3	3.9	0.9929	−70.2	2.9	0.9949	−61.7	4.3
23	Diethylcarbamazine	0.9878	−87.2	0.2	0.9931	−41.7	0.5	0.9983	−91.7	1.2
24	Doramectin	0.9946	−33.9	0.2	0.9976	98.4	0.03	0.9871	55.7	0.3
25	Emamectin	0.9803	−66.5	0.3	0.9948	−34.7	0.3	0.9986	-19.4	0.1
26	Ethopabate	0.9999	−44.6	0.1	0.9988	−1.0	0.2	0.9984	−5.5	0.1
27	Febantel	0.9938	−55.3	0.2	0.9973	−27.6	0.1	0.9992	−11.8	0.1
28	Fenbendazole	0.9846	−72.0	0.5	0.9978	−52.4	0.5	0.9920	−32.8	0.3
29	Fluazuron	0.9983	−80.9	0.4	0.9956	−74.7	0.7	0.9960	−97.4	0.2
30	Flubendazole	0.9984	−44.6	0.1	0.9996	−13.3	0.1	0.9965	−4.9	0.1
31	2-Amino flubendazole	0.9894	−56.8	0.2	0.9918	−61.6	0.2	0.9988	−18.0	0.2
32	Halofuginone	0.9970	−58.7	0.1	0.9893	−30.0	0.1	0.9959	−30.6	0.2
33	Imidocarb	0.9881	−82.8	1.6	0.9941	−37.6	0.6	0.9907	−84.3	2.5
34	Ivermectin	0.9840	−49.7	1.0	0.9972	−35.4	0.5	0.9966	31.4	3.0
35	Levamisole	0.9992	−44.0	0.4	0.9863	−24.2	0.4	0.9997	−31.4	2.1
36	Maduramycin	0.9992	−68.9	0.5	0.9846	−56.4	0.4	0.9972	−24.6	0.1
37	Mebendazole	0.9986	−48.3	0.04	0.9952	−6.4	0.1	0.9970	29.8	0.2
38	Mebendazole amine	0.9978	−48.3	0.3	0.9952	−38.5	0.3	0.9983	−22.5	0.1
39	5-Hydroxy mebendazole	0.9995	−44.6	0.9	0.9986	−45.9	0.9	0.9991	−3.1	1.1
40	Methylbenzoquate, Nequinate	0.9993	−60.0	0.1	0.9961	−79.0	0.1	0.9997	−13.0	0.1
41	Metronidazole	0.9942	−54.5	0.3	0.9977	−28.0	0.2	0.9995	−48.7	0.5
42	Monensin	0.9874	−53.4	0.7	0.9866	−55.9	0.2	0.9951	−29.0	0.1
43	Monepantel	0.9871	92.4	0.3	0.9985	106.4	0.1	0.9966	25.5	0.1
44	Monepantel-sulfone	0.9884	49.7	0.2	0.9910	108.7	0.1	0.9876	22.1	0.04
45	Morantel	0.9997	−34.8	0.3	0.9989	−3.0	0.9	0.9997	−14.8	0.4
46	Narasin	0.9911	−75.4	0.3	0.9914	−80.1	0.4	0.9982	−48.2	0.2
47	Nicarbazin	0.9862	−30.7	0.2	0.9911	−36.4	0.1	0.9952	−15.9	0.2
48	Niclosamide	0.9990	−47.6	0.2	0.9833	−57.4	0.1	0.9995	−56.0	0.1
49	Nitroxinil	0.9907	−37.7	0.1	0.9883	−7.0	0.2	0.9806	−5.8	0.1
50	Ornidazole	0.9985	−44.4	0.4	0.9997	−18.2	0.6	0.9958	−9.8	0.3
51	Oxamniquine	0.9988	−60.5	0.1	0.9991	−15.8	0.8	0.9996	−31.5	0.8
52	Oxantel	0.9982	−30.2	0.5	0.9985	−10.3	0.3	0.9992	−13.0	0.1
53	Oxfendazole	0.9988	−33.0	3.6	0.9998	−7.7	4.3	0.9971	−13.1	2.4
54	Oxfendazole sulfone	0.9979	−23.1	0.1	0.9963	−0.1	0.03	0.9987	−4.3	0.1
55	Oxibendazole	0.9929	−37.9	1.2	0.9971	−32.8	0.5	0.9989	−18.1	1.2
56	Oxyclozanide	0.9837	−10.2	1.8	0.9919	−15.9	1.5	0.9857	−11.7	1.0

57	Praziquantel	0.9972	−46.0	0.1	0.9995	−8.2	0.2	0.9994	−1.3	0.2
58	Pyrantel	0.9999	−29.8	0.2	0.9994	−7.0	0.2	0.9999	−11.7	0.2
59	Salinomycin	0.9930	−74.1	0.1	0.9981	−72.2	0.1	0.9974	−48.9	0.1
60	Semduramicin	0.9894	−37.8	0.5	0.9850	−45.5	0.2	0.9966	9.7	0.1
61	Ternidazole	0.9955	−57.0	1.4	0.9967	−14.1	0.7	0.9976	−22.7	1.2
62	Tetramisole	0.9998	−36.4	0.2	0.9988	−22.3	0.3	0.9964	−36.0	0.2
63	Thiabendazole	0.9940	−44.2	0.1	0.9944	−17.8	0.1	0.9949	−29.8	0.1
64	5-Hydroxy thiabendazole	0.9983	−78.8	0.1	0.9982	−80.1	0.2	0.9997	−76.7	0.1
65	Thiophanate	0.9893	−71.1	0.2	0.9982	−63.6	0.1	0.9987	−44.7	0.1
66	Tinidazole	0.9956	−34.7	2.1	0.9966	−25.8	2.1	0.9936	−29.6	1.9
67	Toltrazuril sulfone	0.9963	−32.6	0.1	0.9991	−11.7	0.1	0.9992	−4.4	0.4
68	Trichlorfon	0.9879	−67.3	1.6	0.9964	−17.0	0.6	0.9951	24.4	1.5
69	Triclabendazole	0.9994	−79.5	0.2	0.9968	−68.5	0.7	0.9974	−35.4	0.3
70	Keto triclabendazole	0.9990	−57.7	0.4	0.9846	−58.6	0.3	0.9944	−17.9	0.2
71	Zoalene	0.9981	−16.8	0.3	0.9948	−4.2	0.3	0.9879	−12.8	0.3

[a]Matrix effects, [b] Limit of quantitation.

Table S3. Validation results for the analytical method of 71 compounds in 4 kinds of food matrices (n=5).

No.	Compound	Recovery (CV[a]), %											
		**Flatfish**			**Eel**			**Shrimp**			**Manila clam**		
		**C1[b]**	**C2[b]**	**C3[b]**	**C1[b]**	**C2[b]**	**C3[b]**	**C1[b]**	**C2[b]**	**C3[b]**	**C1[b]**	**C2[b]**	**C3[b]**
1	Abamectin	112.2 (20.5)	104.6 (11.1)	99.8 (13.4)	77.9 (21.4)	109.0 (19.7)	103.7 (5.4)	103.0 (15.2)	100.1 (13.7)	106.6 (11.2)	87.7 (29.7)	106.2 (24.4)	107.3 (10.0)
2	Albendazole	89.0 (8.9)	104.1 (3.9)	95.6 (6.4)	89.0 (10.0)	104.1 (3.7)	95.6 (6.7)	80.0 (9.0)	95.8 (1.3)	101.0 (6.7)	111.2 (4.6)	114.7 (2.8)	107.9 (5.6)
3	Albendazole sulfoxide	111.8 (8.2)	102.7 (3.3)	96.6 (4.2)	99.1 (10.4)	104.3 (10.2)	101.2 (7.2)	110.4 (3.0)	107.6 (3.2)	98.4 (1.8)	116.2 (6.4)	103.8 (10.6)	92.7 (5.2)
4	Albendazole sulfone	112.1 (4.5)	99.9 (3.3)	91.7 (3.5)	110.9 (8.2)	107.1 (1.5)	101.4 (4.2)	97.1 (11.2)	110.4 (1.7)	112.5 (4.5)	109.6 (7.0)	101.2 (6.2)	102.3 (5.3)
5	2-Amino albendazole sulfone	112.1 (4.5)	99.9 (3.3)	91.7 (3.5)	110.9 (8.2)	107.1 (1.5)	101.4 (4.2)	97.1 (11.2)	110.4 (1.7)	112.5 (4.5)	109.6 (7.0)	101.2 (6.2)	102.3 (5.3)
6	Arprinocid	97.5 (4.6)	97.0 (2.5)	97.3 (1.2)	94.5 (8.5)	98.1 (4.0)	97.3 (2.6)	92.8 (4.7)	99.9 (6.1)	98.7 (1.9)	97.9 (2.6)	109.1 (7.3)	107.3 (4.9)
7	Amprolium	115.5 (19.6)	100.2 (7.8)	91.2 (12.1)	103.1 (7.9)	88.1 (3.6)	93.5 (6.0)	109.5 (6.0)	97.9 (14.1)	100.1 (4.8)	107.0 (9.9)	90.2 (4.4)	91.5 (7.3)
8	Benznidazole	90.6 (8.5)	103.8 (3.3)	103.5 (4.6)	94.2 (4.2)	95.5 (5.6)	96.2 (3.4)	95.6 (0.8)	93.8 (3.7)	97.9 (5.1)	91.4 (6.9)	98.8 (3.8)	101.1 (5.7)
9	Bithionol	96.0 (2.7)	105.4 (2.9)	101.5 (2.8)	105.8 (29.8)	93.4 (8.4)	95.0 (19.6)	102.0 (8.4)	104.5 (7.2)	98.0 (17.3)	117.7 (7.1)	117.7 (8.5)	119.4 (2.7)
10	Buparvaquone	99.2 (2.5)	109.0 (2.7)	108.3 (2.6)	110.4 (25.8)	114.2 (23.2)	87.9 (19.9)	105.5 (10.5)	102.4 (3.1)	96.3 (5.1)	107.1 (20.7)	94.4 (19.8)	102.9 (12.5)
11	Buquinolate	88.7 (9.5)	94.8 (15.3)	84.6 (9.6)	93.1 (10.2)	95.6 (17.9)	112.4 (10.5)	90.1 (2.6)	103.8 (2.9)	99.7 (4.5)	107.8 (2.7)	113.4 (3.7)	114.8 (3.5)
12	Cambendazole	108.6 (2.8)	102.8 (4.9)	101.5 (3.0)	83.9 (4.2)	95.8 (4.8)	98.7 (2.2)	91.7 (7.6)	95.6 (4.2)	97.4 (1.0)	95.0 (3.8)	99.2 (3.2)	97.9 (1.7)
13	Carbendazim	95.0 (4.7)	92.1 (6.6)	92.0 (1.8)	103.1 (7.8)	102.3 (5.3)	99.1 (5.8)	105.0 (8.0)	102.2 (4.6)	90.0 (3.8)	104.5 (8.4)	87.4 (4.4)	107.9 (2.0)
14	Carnidazole	106.7 (7.3)	101.9 (1.7)	106.2 (0.8)	92.7 (4.8)	96.4 (3.4)	95.9 (2.5)	95.4 (8.7)	103.2 (3.0)	95.3 (4.5)	84.1 (7.9)	85.1 (13.8)	106.1 (8.5)
15	Clopidol	83.9 (7.0)	95.7 (3.0)	101.3 (3.7)	84.8 (6.4)	97.4 (4.1)	99.6 (7.8)	97.2 (5.1)	99.8 (1.5)	98.8 (2.4)	94.6 (8.0)	96.1 (10.0)	103.7 (5.0)
16	Clorsulon	101.5 (23.5)	94.3 (7.1)	100.0 (5.5)	94.9 (16.9)	92.9 (18.9)	96.9 (6.0)	97.9 (12.1)	97.3 (9.2)	97.8 (7.6)	82.7 (15.8)	78.2 (7.5)	88.0 (6.3)

17	Closantel	84.6 (28.3)	117.4 (22.9)	111.9 (11.8)	101.5 (18.1)	96.0 (7.8)	100.3 (13.6)	112.2 (2.9)	104.6 (4.5)	97.8 (9.2)	74.4 (23.0)	77.3 (27.5)	89.8 (19.3)
18	Cymiazole	92.9 (9.8)	98.7 (8.5)	105.3 (5.1)	86.8 (6.6)	93.8 (6.1)	94.5 (3.3)	95.1 (5.4)	97.7 (3.2)	90.3 (7.2)	111.3 (4.7)	113.5 (2.6)	105.8 (5.1)
19	Decoquinate	85.5 (5.1)	77.5 (7.4)	81.1 (6.1)	99.4 (16.5)	86.0 (22.9)	113.1 (19.7)	76.4 (23.2)	100.3 (7.7)	102.2 (9.4)	109.5 (9.7)	99.7 (6.0)	107.1 (10.0)
20	Diaveridine	96.9 (4.0)	99.1 (3.2)	100.9 (1.6)	91.3 (2.7)	101.1 (3.4)	102.0 (3.4)	87.4 (6.9)	97.2 (7.3)	103.4 (1.9)	95.9 (1.7)	100.7 (3.9)	106.7 (7.7)
21	Diclazuril	87.5 (26.6)	107.7 (4.4)	100.3 (5.6)	67.4 (16.6)	101.3 (11.8)	98.6 (16.1)	76.6 (20.7)	96.9 (1.7)	98.4 (5.3)	107.5 (7.4)	114.4 (7.4)	106.2 (7.3)
22	Dicyclanil	95.9 (17.1)	97.8 (20.2)	103.3 (3.0)	118.0 (13.0)	112.9 (7.5)	110.7 (7.2)	104.8 (13.0)	93.9 (5.1)	96.2 (4.9)	101.8 (8.7)	85.2 (4.7)	104.3 (8.4)
23	Diethylcarbamazine	103.2 (13.0)	88.3 (8.1)	89.5 (9.4)	114.9 (8.4)	107.4 (3.3)	91.7 (1.7)	104.2 (4.1)	96.1 (9.7)	97.4 (12.6)	92.5 (2.8)	98.7 (6.7)	103.7 (5.0)
24	Doramectin	91.4 (16.5)	117.3 (11.7)	114.4 (14.6)	82.2 (25.3)	109.3 (22.1)	117.8 (13.2)	113.9 (26.6)	103.7 (6.1)	109.2 (6.5)	99.1 (7.4)	106.0 (18.0)	107.3 (11.0)
25	Emamectin	85.7 (13.0)	90.4 (8.9)	86.4 (9.6)	87.7 (12.2)	107.9 (16.4)	110.9 (9.6)	90.5 (5.9)	108.3 (4.2)	102.4 (4.2)	90.4 (11.7)	100.6 (9.6)	108.6 (4.9)
26	Ethopabate	103.4 (4.9)	102.7 (1.8)	102.4 (3.0)	112.9 (7.7)	108.4 (4.7)	99.0 (6.3)	101.7 (7.5)	101.8 (2.6)	94.2 (4.4)	105.4 (2.7)	106.6 (2.9)	105.3 (2.3)
27	Febantel	93.1 (12.5)	103.7 (4.7)	92.6 (6.3)	86.8 (13.6)	94.8 (6.3)	93.5 (4.3)	98.8 (4.1)	100.5 (8.3)	100.5 (4.7)	80.1 (12.1)	92.9 (21.3)	108.9 (4.9)
28	Fenbendazole	113.2 (2.3)	110.0 (5.4)	93.5 (6.7)	108.3 (10)	105.2 (14)	101.6 (11.7)	80.7 (4.5)	103.8 (2.7)	92.8 (17.5)	75.3 (27.3)	88.7 (15.7)	103.0 (4.1)
29	Fluazuron	89.9 (12.7)	100.8 (9.5)	97.8 (7.1)	105.1 (15.4)	97.4 (7.8)	104.4 (15.2)	99.1 (10.2)	97.3 (3.0)	103.7 (10.3)	118.6 (16.0)	93.3 (9.2)	68.9 (7.4)
30	Flubendazole	100.9 (4.8)	105.3 (2.5)	93.4 (10.4)	106.1 (8.9)	100.4 (7.1)	86.3 (4.0)	89.6 (7.8)	100.1 (6.4)	90.9 (6.0)	109.6 (7.4)	118.6 (6.3)	106.5 (5.9)
31	2-Amino flubendazole	96.8 (5.2)	99.1 (6.9)	101.0 (5.7)	80.4 (20.3)	104.8 (9.9)	100.5 (14.1)	101.7 (2.7)	104.8 (7.5)	97.2 (3.7)	113.6 (4.7)	108.4 (3.7)	109.8 (5.2)
32	Halofuginone	111.8 (7.9)	108.3 (7.7)	97.4 (8.3)	105 (16.3)	107.9 (8.5)	97.0 (4.8)	108.7 (1.2)	97.0 (4.3)	94.7 (9.5)	106.7 (11.1)	99.0 (11.0)	109.7 (8.4)
33	Imidocarb	96.6 (8.2)	90.7 (10.1)	77.4 (11.6)	76.8 (15.7)	85.8 (5.9)	108.6 (2.3)	107.0 (9.3)	95.6 (12.1)	107.2 (7.2)	114.3 (5.2)	110.5 (12.5)	108.4 (6.8)
34	Ivermectin	112.8 (12.5)	102.5 (8.3)	100.5 (12.3)	117.1 (15.5)	105.5 (15.6)	107.0 (20.4)	83.1 (24.6)	93.1 (10.4)	94.2 (7.1)	93.8 (14.2)	117.6 (8.9)	106.9 (4.1)
35	Levamisole	109.7 (2.7)	102.5 (5.8)	97.7 (2.6)	96.8 (12.5)	93.9 (1.9)	94.0 (2.5)	100.0 (3.6)	98.8 (2.5)	86.2 (2.7)	94.9 (3.0)	110.5 (6.6)	111.3 (8.6)
36	Maduramycin	108.3 (20.2)	109.1 (23.1)	93.4 (17.9)	112.8 (28.6)	116.6 (10.2)	87.3 (16.5)	71.6 (22.7)	102.6 (8.7)	94.1 (3.9)	86.9 (9.7)	115.8 (13.9)	111.1 (8.3)
37	Mebendazole	106.1 (3.4)	95.1 (3.7)	98.9 (5.0)	84.7 (10.6)	95.7 (2.0)	97.6 (6.6)	95.1 (5.7)	96.8 (7.0)	98.0 (9.2)	105.0 (3.2)	102.4 (5.1)	104.5 (2.9)
38	Mebendazole amine	90.9 (8.6)	103.3 (1.6)	95.2 (3.1)	99.7 (8.9)	89.6 (11.9)	100.4 (5.1)	84.1 (5.9)	86.4 (10.8)	95.6 (4.1)	95.0 (12.7)	100.2 (5.7)	105.8 (8.4)
39	5-Hydroxy mebendazole	94.2 (9.0)	101.5 (1.9)	101.4 (2.2)	103.2 (3.9)	95.3 (3.5)	94.0 (2.0)	95.6 (6.4)	101.5 (4.4)	97.1 (2.6)	101.0 (2.9)	95.5 (8.0)	104.1 (4.1)
40	Methylbenzoquate, Nequinate	96.7 (14.4)	110.0 (14.4)	106.2 (9.6)	96.9 (18.6)	119.4 (19.6)	115.3 (14.8)	97.5 (8.2)	102.7 (4.3)	101.7 (1.6)	101.1 (10.4)	111.1 (8.9)	108.3 (12.8)
41	Metronidazole	117.4 (9.4)	104.1 (14.7)	99.6 (6.9)	96.4 (11.3)	102.2 (4.4)	90.7 (4.4)	108.6 (9.9)	99.5 (8.4)	100.8 (15.9)	86.1 (13.6)	102.5 (11.7)	86.7 (11.8)
42	Monensin	93.5 (19.1)	106.5 (12.7)	105.6 (12.1)	98.4 (24.4)	110.2 (12.4)	107.1 (16.6)	78.9 (15.1)	98.8 (4.9)	105.7 (5.6)	107.1 (12.2)	93.1 (14.0)	114.9 (4.7)
43	Monepantel	104.4 (19.8)	113.4 (11.1)	114.4 (9.9)	92.5 (10.7)	101.8 (11)	102.8 (6.6)	79.4 (12.1)	92.4 (5.9)	96.6 (8.1)	77.0 (14.7)	94.3 (16.2)	112.6 (6.9)
44	Monepantel-sulfone	97.3 (9.4)	107.3 (3.6)	104.2 (5.9)	81.1 (6.8)	107.0 (2.5)	110.9 (8.9)	69.5 (10.5)	98.9 (5.5)	103.6 (10.8)	89.3 (2.0)	98.2 (7.9)	109.7 (4.2)
45	Morantel	90.9 (4.1)	101.3 (3.7)	98.4 (2.4)	101.5 (3.2)	99.1 (2.4)	92.8 (1.5)	100.5 (5.0)	99.9 (4.7)	96.8 (1.0)	88.9 (6.6)	94.8 (5.9)	101.4 (5.3)
46	Narasin	100.4 (7.4)	113.9 (20.3)	108.7 (17.6)	101.0 (30.2)	99.4 (24.0)	87.4 (20.3)	84.0 (19.1)	93.9 (8.6)	105.7 (5.4)	82.9 (29.5)	88.0 (23.5)	103.7 (9.5)

47	Nicarbazin	90.4 (8.3)	101.8 (6.4)	88.7 (6.0)	109.1 (7.7)	93.2 (10.2)	110.2 (9.9)	107.9 (5.6)	114.2 (4.3)	101.4 (10.6)	109.3 (10.6)	87.5 (8.2)	110.1 (11.7)
48	Niclosamide	103.6 (18.0)	119.5 (8.4)	99.4 (7.2)	108.6 (24.3)	93.4 (16.9)	94.8 (14.3)	117.0 (7.1)	118.8 (4.8)	114.4 (9.0)	87.6 (11.5)	115.2 (26.0)	112.6 (4.8)
49	Nitroxinil	84.4 (3.3)	110.7 (2.2)	114.9 (12.3)	68.4 (3.7)	102.8 (3.0)	111.1 (6.8)	78.3 (5.2)	106.5 (3.3)	92.4 (1.6)	72.1 (8.3)	94.1 (10.5)	113.0 (6.1)
50	Ornidazole	99.6 (9.1)	98.8 (5.9)	97.2 (5.8)	93.7 (15.8)	89.4 (11.0)	94.9 (5.4)	101.0 (18)	101.5 (8.9)	92.4 (8.7)	117.9 (10.2)	106.5 (5.7)	101.2 (5.9)
51	Oxamniquine	91.5 (7.3)	101.4 (2.2)	102.6 (2.5)	105.4 (5.7)	100.3 (5.2)	92.3 (5.7)	94.7 (6.6)	105.5 (3.1)	96.7 (3.0)	104.4 (12.5)	105.6 (6.1)	105.5 (8.0)
52	Oxantel	95.1 (3.5)	98.0 (2.8)	100.7 (2.4)	93.9 (4.6)	97.8 (3.2)	99.6 (4.8)	97.1 (2.0)	104.5 (2.6)	102.7 (1.6)	100.4 (2.6)	97.6 (5.9)	104.4 (1.8)
53	Oxfendazole	100.7 (9.6)	95.7 (2.8)	97.7 (3.3)	109.8 (4.7)	101.7 (4.2)	100.6 (2.7)	89.0 (3.9)	95.3 (1.1)	96.6 (3.6)	116.0 (6.2)	99.5 (6.6)	98.6 (4.1)
54	Oxfendazole sulfone	118.9 (4.4)	106.9 (1.6)	95.6 (5.0)	110.8 (4.2)	101.6 (4.1)	102.1 (1.9)	93.6 (3.4)	100.6 (4.8)	94.7 (4.6)	96.1 (2.5)	98.6 (8.9)	99.6 (5.2)
55	Oxibendazole	74.5 (22.3)	118.7 (4.8)	119.0 (4.0)	83.8 (10.6)	107.1 (2.1)	105.7 (6.4)	94.0 (5.0)	99.1 (5.2)	94.9 (2.8)	98.8 (6.0)	100.6 (4.3)	110.4 (4.0)
56	Oxyclozanide	80.9 (18.2)	110.2 (11.5)	111.7 (9.8)	60.7 (6.0)	102.1 (12.6)	96.3 (13.4)	68.6 (24.3)	105.1 (1.3)	101.5 (11.2)	91.6 (9.6)	115.9 (11.5)	98.4 (11.3)
57	Praziquantel	105.5 (7.1)	106.4 (2.4)	102.6 (4.5)	101.6 (8.8)	100.9 (4.8)	107.0 (5.4)	95.6 (1.4)	96.7 (4.9)	95.4 (2.5)	109.3 (2.7)	103.4 (11.5)	110.2 (2.8)
58	Pyrantel	97.7 (4.3)	96.4 (2.1)	93.6 (3.0)	111.9 (4.4)	101.5 (4.5)	96.3 (3.7)	97.8 (4.0)	98.9 (2.3)	93.2 (2.7)	102.5 (3.3)	102.2 (2.1)	103.1 (2.9)
59	Salinomycin	117.5 (5.3)	113.5 (14.3)	107.1 (18.8)	96.7 (28.6)	100.2 (17.7)	80.1 (17.6)	78.7 (26.3)	91.9 (7.6)	109.2 (7.3)	66.9 (8.8)	85.5 (27.0)	101.4 (16.7)
60	Semduramicin	79.8 (16.8)	108.0 (18.3)	99.8 (15.3)	88.6 (19.4)	104.0 (14.4)	113.0 (15.4)	74.0 (12.9)	95.4 (6.4)	101.2 (3.7)	95.9 (15.2)	113.3 (19.7)	113.1 (3.9)
61	Ternidazole	98.4 (8.6)	97.6 (2.5)	102.6 (4.8)	88 (7.5)	101.7 (7.0)	101.3 (10.3)	101.2 (7.0)	110.9 (4.7)	100.6 (6.3)	85.8 (19.6)	99.5 (5.2)	99.6 (3.4)
62	Tetramisole	106.6 (2.6)	101.4 (3.7)	101.0 (4.5)	104.9 (3.4)	103.7 (3.2)	96.3 (4.3)	84.5 (4.4)	92.3 (3.2)	91.6 (8.6)	84.8 (5.4)	109.3 (8.2)	110.7 (7.3)
63	Thiabendazole	78.6 (8.8)	92.7 (5.7)	100.0 (5.7)	67.0 (10.7)	91.5 (4.0)	98.6 (5.3)	77.1 (6.6)	94.4 (3.3)	99.2 (3.4)	102.6 (4.9)	102.6 (5.1)	103.8 (3.5)
64	5-Hydroxy thiabendazole	117.1 (17.6)	101.4 (12.2)	96.8 (15.5)	85.1 (17.2)	106.3 (7.2)	100.2 (6.7)	104.2 (7.6)	99.1 (8.6)	110.6 (6.0)	83.3 (4.1)	105.3 (8.3)	102.7 (10.9)
65	Thiophanate	93.5 (14.3)	119.6 (5.5)	119.9 (17.8)	86.1 (17.9)	70.7 (24.6)	73.9 (12.7)	101.9 (8.1)	91.0 (12.9)	85.6 (8.6)	86.1 (9.7)	86.1 (21.1)	104.3 (16.2)
66	Tinidazole	99.1 (18.0)	86.0 (11.2)	86.6 (6.4)	84.1 (12.9)	92.0 (9.5)	100.8 (4.5)	101.1 (14.0)	85.3 (13.3)	97.8 (7.9)	107.1 (19.0)	99.9 (4.3)	95.1 (8.8)
67	Toltrazuril sulfone	86.2 (9.4)	98.7 (9.0)	104.2 (4.6)	101.1 (5.4)	103.6 (11.3)	102.8 (4.5)	93.1 (14.4)	100.4 (1.6)	95.6 (3.7)	89.7 (4.7)	101.6 (14.4)	103.5 (6.8)
68	Trichlorfon	106.0 (6.9)	94.3 (8.3)	97.4 (10.2)	81.2 (19.2)	87.6 (11.9)	89.6 (8.3)	104.9 (13.7)	91.1 (5.8)	94.0 (3.9)	112.3 (9.6)	101.3 (12.6)	104.7 (9.7)
69	Triclabendazole	114.8 (10.9)	117.0 (7.6)	108.4 (11.9)	92.2 (8.7)	108.2 (12.8)	101.7 (8.8)	97.0 (11.8)	112.2 (10.5)	108.6 (9.1)	110.4 (24.9)	106.9 (20.7)	116.6 (22.2)
70	Keto triclabendazole	98.3 (27.6)	118.9 (8.9)	106.2 (14.3)	60.5 (27.8)	102.8 (18.2)	99.9 (19.0)	88.2 (20.6)	95.5 (7.3)	96.1 (6.5)	105.1 (21.2)	96.0 (12.3)	108.7 (8.0)
71	Zoalene	101.8 (6.1)	113.1 (7.7)	110.1 (16.9)	108.1 (5.6)	96.8 (8.3)	86.7 (17.6)	90.1 (17.8)	100.9 (14.2)	103.5 (9.7)	119.4 (15.5)	93.2 (18.2)	101.6 (6.8)

[a]Coefficient value (CV) [b]Validation level C1, C2 and C3: 1, 2 and 10 μg kg^{-1} for metronidazole and 5, 10 and 20 μg kg^{-1} for all other analytes.

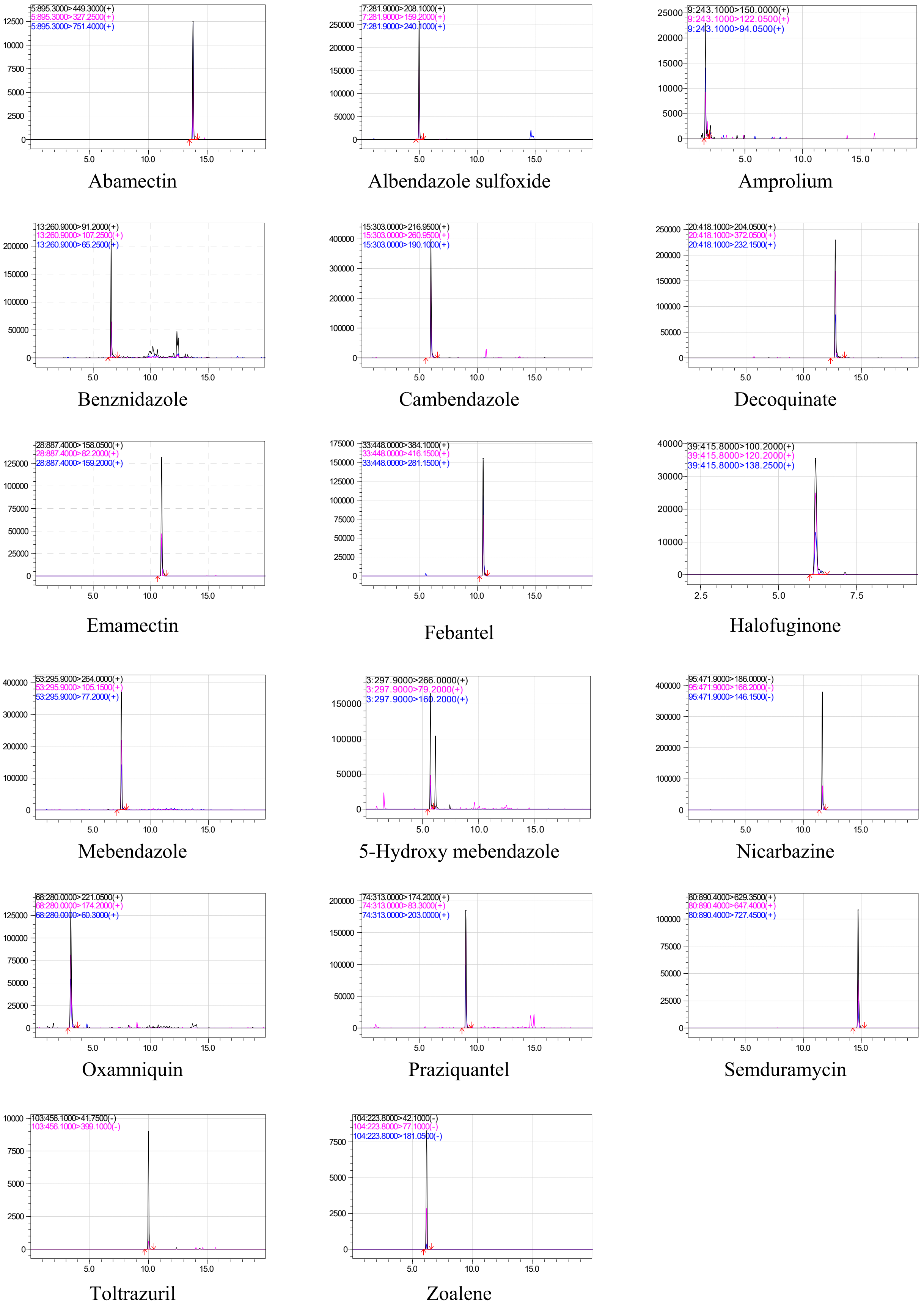

Figure S1. Representative chromatograms in flatfish at the spiking levels of 10 μg kg^{-1}.
